# Ist eine Verbesserung der Behandlungssicherheit in der Korsettversorgung von Skoliosepatienten durch Anwendung standardisierter CAD-Algorithmen möglich?

**DOI:** 10.1007/s00132-020-04000-9

**Published:** 2020-10-06

**Authors:** Hans-Rudolf Weiss, Manuel Lay, Sarah Seibel, Alexander Kleban

**Affiliations:** 1 Gesundheitsforum Nahetal, Alzeyer Str. 23, 55457 Gensingen, Deutschland; 2 Haarbergweg 2, 55546 Neu Bamberg, Deutschland; 3 Orthopädie-Technik Lay, Straße von Triptis 8, 56856 Zell-Barl, Deutschland; 4 Lomonosov Moscow State University, 119234, Leninskie Gory 1, Moskau, Russland

**Keywords:** Rumpforthesen, Mädchen, Wachstum, Computer-aided design, Computer-aided manufacturing, Trunk orthoses, Girls, Growth, Computer-aided design, Computer-aided manufacturing

## Abstract

**Hintergrund:**

Die Versorgung von Skoliosepatienten mit korrigierenden Rumpforthesen führt auch heutzutage noch zu recht unterschiedlichen Ergebnissen. Publizierte Erfolgsquoten zwischen 50 und 90 % führen zwangsläufig zu der Frage, wie sich die Erfolgsquoten der Korsettversorgung steigern und vereinheitlichen lassen. Die Ergebnisse einer mit dieser Zielsetzung weiterentwickelten computerunterstützen (CAD/„Computer Aided Design“) Chêneau-Versorgung werden dargestellt.

**Methodik:**

Am Stichtag (08.12.2019) wurde die prospektiv angelegte Datenbank unserer Abteilung retrospektiv ausgewertet. Es waren ausschließlich unreife Mädchen mit einer Adoleszentenskoliose, Alter 10–14 Jahre, Risser 0–2 in die Datenbank aufgenommen worden.

**Ergebnisse:**

Sowohl die Gesamtgruppe mit einem Beobachtungszeitraum von mindestens 18 Monaten als auch die Patientinnengruppen mit bereits erreichtem Behandlungsabschluss zeigten Erfolgsraten zwischen 86 und 88 %. Die Ergebnisse insgesamt waren signifikant besser als die Erfolgsrate der Boston-Brace-Kontrollgruppe (BRAIST) von 72 %. Auch im Vergleich mit den Ergebnissen anderer Chêneau-Derivate war die Erfolgsrate unserer Serie teils deutlich besser.

**Schlussfolgerungen:**

Die Behandlungssicherheit für die Patienten mit Skoliosen sollte verbessert werden. Ein Ansatz hierzu kann die Verwendung standardisierter CAD-Bibliotheken sein. Weitere Untersuchungen mit Studiendesigns höherer Evidenz sind notwendig, um die in unserer Untersuchung gefundenen Ergebnisse zu untermauern.

## Abstract (en)

**Background:**

The treatment of scoliosis patients with corrective braces today still leads to quite different results. The published success rates between 50 and 90% inevitably lead to the question of how the success rates of brace treatment can be increased and standardized. The results of a computer aided design (CAD) Chêneau application, developed with this objective, are presented.

**Materials and methods:**

On the reporting date (December 8, 2019), the prospectively created database of our department was evaluated retrospectively. Only immature girls with adolescent idiopathic scoliosis (AIS), aged 10–14 years, Risser signs 0–2, were included in the database.

**Results:**

Both the entire group with an observation period of at least 18 months and the patient groups with completed treatment had success rates between 86 and 88%. Overall, the results were significantly better than the 72% success rate of the Boston Brace Control Group (BRAIST). In comparison with the results of other Chêneau derivatives, the success rate of our series was distinctly better.

**Conclusions:**

Treatment safety in brace treatment for patients with scoliosis should be improved. One approach may be the use of standardized CAD libraries. Further investigations with study designs of higher evidence are necessary to support the results found in our investigation.

Die Versorgung von Skoliosepatienten mit korrigierenden Rumpforthesen führt auch heutzutage noch zu recht unterschiedlichen Ergebnissen. Publizierte Erfolgsquoten zwischen 50 und 90 % führen zwangsläufig zu der Frage, wie sich die Erfolgsquoten der Korsettversorgung steigern und vereinheitlichen lassen. Schließlich beeinträchtigt jedwede Korsettversorgung die Lebensqualität der betroffenen Patienten. Daher sollten alle Anstrengungen unternommen werden, die Korsettversorgung sicherer und gleichzeitig bequemer zu machen. Die Ergebnisse einer mit dieser Zielsetzung weiterentwickelten CAD-Chêneau-Versorgung werden dargestellt.

## Einleitung

Skoliosen sind dreidimensionale Deformitäten von Rumpf und Wirbelsäule, welche sich in Phasen verstärkten Wachstums drastisch verschlechtern können [1, 10, 15]. Es gibt eine Vielzahl von Ursachen für die Entstehung derartiger Wirbelsäulenverkrümmungen (kongenitale Skoliosen mit Fehlbildungen von Wirbelkörpern und Rippen, neuromuskuläre Skoliosen, Skoliosen bei mesenchymalen Defekten und vielen anderen Grunderkrankungen und Syndromen) [6]. Den Hauptteil aller Skoliosen stellen allerdings die Skoliosen mit unbekannter Ursache (idiopathische Skoliosen), von welchen die idiopathischen Adoleszentenskoliosen insgesamt mit ca. 80 % am häufigsten auftreten. Daher gibt es zu dieser Skolioseform auch die meisten wissenschaftlichen Untersuchungen [1, 10, 15].

Die idiopathische Adoleszentenskoliose tritt während des puberalen Wachstumsschubes auf und betrifft vorzugsweise Mädchen (weiblich zu männlich ca. 4 : 1), bei Krümmungen mit einem Krümmungswinkel von 40° und mehr ist das Geschlechterverhältnis noch weiter zu Ungunsten der Mädchen verschoben (weiblich zu männlich ca. 10 : 1) [1, 10, 15].

Die Behandlung der idiopathischen Skoliose besteht aus:

- Verlaufsbeobachtung
- Physiotherapie
- Korsettversorgung
- Operation

Die Indikationsleitlinien zur konservativen Skoliosebehandlung sind erstmals 2006 veröffentlicht worden [33] und haben sich seit dem nur unwesentlich geändert [41].

Im Hauptwachstumsschub kann die Progressionswahrscheinlichkeit nach Lonstein und Carlson für den Einzelfall errechnet werden [18]. Ab einer Progressionswahrscheinlichkeit von 60 % besteht eine Korsettindikation [33, 41].

Die Korsettversorgung von Kindern und Adoleszenten kann heutzutage als evidenzbasierte Maßnahme angesehen werden [23, 30, 32]. Allerdings existieren weltweit eine Vielzahl unterschiedlicher Behandlungsansätze und Behandlungsphilosophien. Die Softbrace-Behandlung oder die reine Nachtversorgung im Hauptindikationsbereich (25–40°) haben sich im Grunde nicht bewährt [8, 11, 12, 21, 43]. Die Versorgung mit dem Boston-Brace wurde durch eine randomisierte Studie belegt [30]. Allerdings hat sich in späteren Studien gezeigt, dass Chêneau-basierte Versorgungen zu besseren Endergebnissen führen können [13, 14, 16, 19, 20, 32, 37, 40]. In einem aktuellen Review zur Korsettversorgung von Kindern und Jugendlichen fanden sich jedoch auch bei Chêneau-basierten Korsetten recht unterschiedliche Ergebnisse [4, 13, 14, 16, 19, 20, 24, 32, 37, 40, 44]. Die Erfolgsquoten in den zitierten Studien schwankten zwischen 50 und 90 % [42].

Dies mag zum einen daran liegen, dass immer noch viele Korsette nach Gipsabdruck hergestellt werden. Bei dieser Herstellungsweise ergeben sich kaum Möglichkeiten zur Standardisierung. Die Korsette werden für jeden Patienten individuell neu modelliert und somit kann man nicht auf bereits erfolgreich getesteten und standardisierten Modellen für unterschiedliche Krümmungsmuster aufbauen. CAD/CAM („computer aided design“/„computer aided manufacturing“) kann zur Vereinheitlichung des Versorgungsstandards beitragen. Jedoch bringt nicht die Nutzung der CAD/CAM-Technologie alleine eine Verbesserung der Behandlungsergebnisse.

Dabei zeigen individuell hergestellte Korsette nicht per se schlechtere Ergebnisse als eine CAD-Versorgung. Allerdings können viele Orthopädietechniker wegen ihrer geringen Fallzahl nicht die zur sicheren Versorgung notwendige Expertise entwickeln.

Von Experten überwachte und ständig verbesserte CAD/CAM-Korsettbibliotheken lassen den Korsettstandard insgesamt verbessern. Entsprechende Versorgungen benötigen keine digitalen Nacharbeiten mehr. Bei solch standardisierten Systemen werden Korsettformen für unterschiedliche Krümmungsmuster seit mehr als einem Jahrzehnt weiterentwickelt und qualitätsgesicherte Grundmodelle von erfahrenen CAD-Designern bereits digital and den 3D-Scan des Patienten angepasst, sodass wesentliche Veränderungen durch den Techniker vor Ort nicht vonnöten sind. Die Fähigkeiten und Fertigkeiten zur Feinanpassung entsprechender Modelle an die Patienten werden in der Regel innerhalb weniger Tage in speziell ausgeschriebenen Kursen vermittelt.

In Deutschland ist das Rigo-Chêneau-Korsett und das Regnier-Chêneau-Korsett gut eingeführt. International hat das Gensingen-Brace (GBW) eine recht weite Verbreitung gefunden. Das GBW wurde 2010 eingeführt [35] und wird in Kooperation mit internationalen Spezialisten stetig weiterentwickelt. Zur Versorgung mit dem GWB wurde die auch von Dr. Chêneau verwendete Lehnert-Schroth-Klassifikation (funktionell dreibogig/funktionell vierbogig) zur augmentierten Lehnert-Schroth-Klassifikation mit 7 Grundmustern erweitert [35, 40].

Im Folgenden sind die Grundmuster der augmentierten Lehnert-Schroth-Klassifikation aufgeführt:

- 3BH (großbogige Thorakalkrümmung ohne wesentliche Lumbalkrümmung/Lenke-A-Muster)
- 3BTL (großbogige Thorakalkrümmung ohne wesentliche Lumbalkrümmung/Lenke-A-Muster mit Scheitelwirbel Th 12)
- 3BN (thorakale Hauptkrümmung mit kleinerer und kurzbogiger Lumbalkrümmung/Lenke B)
- 3BL (thorakale Hauptkrümmung mit kleinerer und langbogiger Lumbalkrümmung/Lenke C)
- 4B (Double-major-Muster mit struktureller Thorakal- und Lumbalkrümmung/Lenke 4C/Lenke 6)
- 4BL (lumbale Hauptkrümmung mit kleinerer und kurzbogiger Thorakalkrümmung/Lenke 5)
- 4BTL (thorakolumbale Hauptkrümmung mit kleinerer und kurzbogiger Thorakalkrümmung/Lenke 5, Scheitelwirbel L1)

**Figure 1:**
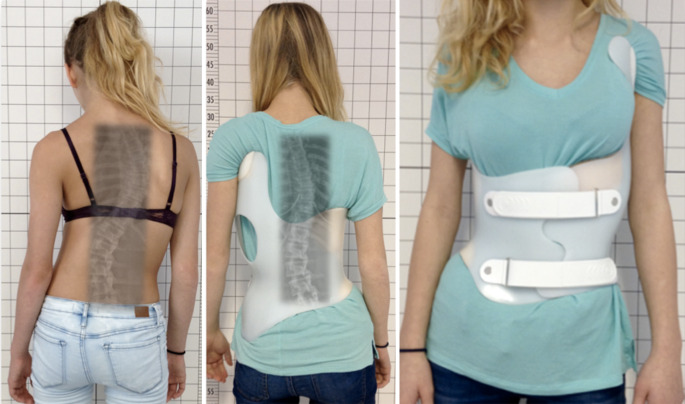

Die der GBW-Versorgung (Abb. 1) zugrunde liegenden CAD-Algorithmen werden für jedes der 7 Grundmuster festgelegt. Die CAD-Korrekturparameter für die einzelnen Krümmungsabschnitte werden für unterschiedliche Krümmungsstärken (20–30°; 30–40°; 40–50° und für Einzelfälle auch darüber hinaus) und unterschiedliche Reifegrade individuell eingestellt. Im Folgenden sollen die in unserer Abteilung mit dem GBW erzielten Ergebnisse dargestellt und mit den Ergebnissen des Boston-Korsetts [30] hinsichtlich der Erfolgsrate („rate of success“) als Zielparameter verglichen werden.

## Material und Methode

Am Stichtag (08.12.2019) wurde die prospektiv angelegte Datenbank unserer Abteilung retrospektiv ausgewertet. Es handelt sich um eine retrospektive Untersuchung (Chart Review) derjenigen Patientinnen, welche die unten aufgeführten Einschlusskriterien erfüllten. Diese Einschlusskriterien für Korsettstudien bei Skoliosen gehen auf Empfehlungen der Scoliosis Research Society (SRS) zurück [25].

Einschlusskriterien zur Aufnahme in die seit 2011 geführte Datenbank waren:

- ausschließlich Mädchen
- Diagnose einer idiopathischen Adoleszentenskoliose
- Alter 10–14 Jahre (bei Erstbeobachtung)
- Risser 0–2
- Krümmungswinkel ab 25 °Cobb

Am Stichtag befanden sich 151 Patientinnen in unserer Datenbank. Ausgewertet wurden folgende Patientinnengruppen:

- Patientinnen mit einem minimalen Follow-up von 18 Monaten (Gruppe 1)
- Endresultate insgesamt (Gruppe II)
- Endresultate mit einem Cobb-Winkel von 25–40° (SRS-Einschlusskriterien/Gruppe III)
- Endresultate mit einem Cobb-Winkel von 40° und darüber (Gruppe IV)

Die Gruppe I ist die Gesamtgruppe aller in diese Untersuchung einbezogener Patientinnen. Diese beinhaltet die Gruppen II–IV als Untergruppen, welche wir einer stärker differenzierten Betrachtung unterziehen wollen.

Die Endresultate wurden 3–36 Monate nach vollständiger Korsettabschulung ermittelt. Die Patientinnen mit einem Krümmungswinkel von mehr als 40° hatten sich ausnahmslos gegen eine Operation und meist für eine Weiterbehandlung in unserer Abteilung entschieden, da in der vorbehandelnden Praxis/Klinik nach stellen der Operationsindikation eine Behandlung mit einem Korsett abgelehnt worden war.

Die 87 Patientinnen der Gruppe I mit einem durchschnittlichen Cobb-Winkel von 40,9° (SD 12,7) hatten einen Beobachtungszeitraum von mindestens 18 Monaten. Das Durchschnittsalter in dieser Gruppe betrug 12,3 Jahre (SD 0,96), die Menarche war durchschnittlich seit 2,1 Monaten aufgetreten (SD 3,6). Das durchschnittliche Risser-Stadium lag bei 0,6 (SD 0,9).

49 % der Patientinnen hatten eine thorakale Hauptkrümmung, 35 % eine Double-major-Krümmung, 8 % eine lumbale und weitere 8 % eine thorakolumbale Hauptkrümmung. Die durchschnittliche Korsetttragezeit wurde mit 20,6 h/Tag (SD 2,9) angegeben. Die Angaben hierzu stammen von den Patientinnen und deren Eltern.

Der durchschnittliche Beobachtungszeitraum aller in dieser Studie untersuchten Patientinnen (Gruppe I) betrug 30,8 Monate (SD 12,3). Der durchschnittliche Beobachtungszeitraum aller in dieser Studie abgeschlossenen Patientinnen (Gruppe II) betrug 36,8 Monate (SD 14,9).

Die Reifecharakteristika und die Cobb-Winkel-Verteilung aller 4 Gruppen sind der Tab. 1 und 2 zu entnehmen. Die Krümmungsmusterverteilung innerhalb der 4 Gruppen ist in der Tab. 3 aufgeführt, wobei die Gruppe I die Gruppen mit den Endresultaten (Gruppen II–IV) beinhaltet.

**Table 1:** 

	*n*	Alter	Menarche	Risser
Gruppe I	87	12,3 (SD 0,96)	2,1 (SD 3,6)	0,6 (SD 0,9)
Gruppe II	39	12,4 (SD 0,99)	2,5 (SD 3,8)	0,7 (SD 0,9)
Gruppe III	24	12,5 (SD 0,98)	3,0 (SD 4,3)	0,8 (SD 0,9)
Gruppe IV	17	12,3 (SD 1,05)	2,5 (SD 3,4)	0,8 (SD 1)

Mittelwerte mit Standardabweichung (SD), Menarche in Monaten seit Erstauftreten

**Table 2:** 

	*n*	Cobb Beginn	Cobb Korsett	Cobb Ende
Gruppe I	87	40,9 (SD 12,7)	21,8 (SD 13,1)	38,2 (SD 16,2)
Gruppe II	39	37,9 (SD 10,8)	19,6 (SD 11)	34,5 (SD 14,5)
Gruppe III	24	30,8 (SD 4,7)	14,8 (SD 7,9)	27,3 (SD 8,8)
Gruppe IV	17	48,2 (SD 7,4)	25,3 (SD 12,3)	44,8 (SD 14,2)

Cobb-Mittelwerte und -Standardabweichung (SD) in den einzelnen Behandlungsgruppen

**Table 3:** 

	*n*	Thorakal (in %)	Double major (in %)	Lumbal (in %)	Thorakolumbal (in %)
Gruppe I	87	49	35	8	8
Gruppe II	39	41	36	13	10
Gruppe III	24	42	29	16	13
Gruppe IV	17	47	41	6	6

Die Progressionsrate (Verschlechterung des Cobb-Winkels 6° und mehr) wurde für alle 4 Gruppen ermittelt, ebenso wie der Prozentsatz der stabilisierten (±5°) und verbesserten Patientinnen (6° und mehr). Die ermittelte Erfolgsrate (stabilisierte + verbesserte Hauptkrümmungen) als Zielparameter unserer Untersuchung wurde mit der Erfolgsrate der BRAIST(Bracing of Adolescent Idiopathic Scoliosis Trial)-Studie von Weinstein und Mitarbeitern [30] verglichen.

### Gensingen-Brace (GBW)

Das GBW ist ein weiterentwickeltes Chêneau-Korsett mit individuellem Design, welches Computer unterstützt (CAD) angepasst werden kann [37, 39]. Die einzelnen Produktionsschritte sind bereits in der Literatur beschrieben [39].

Zunächst wird die Patientin gescannt und es werden die Patientendaten erhoben und gemeinsam mit dem Röntgenbild in die Datenbank eingepflegt. Anhand dieser Daten wird zunächst einmal das dem Krümmungsmuster entsprechende Grundmodell aus der Korsettbibliothek ausgewählt.

**Figure 2:**
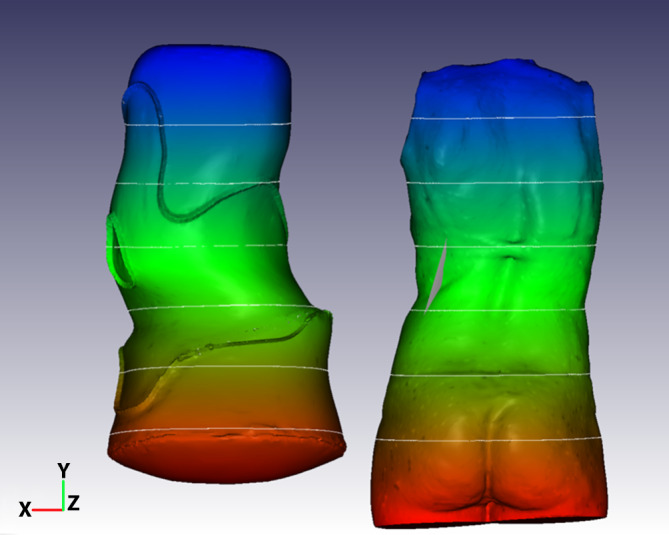

Der Patientenscan wird beschnitten und skaliert. Anschließend wird das ausgewählte Korsett in die Szene eingefügt und entsprechend der individuellen Körperform angepasst. Danach werden entsprechend die für das jeweilige Muster und die jeweilige Krümmungsstärke (Cobb-Winkel) festgelegten Korrekturalgorithmen angewendet. Im Ergebnis erhält man ein Korsettmodell, welches das jeweilige Krümmungsmuster widerspiegelt (Abb. 2).

### Kontrollgruppe

Die randomisierte BRAIST-Untersuchung von Weinstein und Mitarbeitern [30] hat an die SRS-Kriterien für Korsettstudien [25] angelehnte Einschlusskriterien, wodurch Patientinnen definierter Reife und definierter Krümmungswinkel einbezogen werden, was die Vergleichbarkeit erleichtert. Ferner sind die Ergebnisse zum Boston-Korsett relativ homogen (Erfolgsraten um die 70 % sowohl in der SRS-Multicenter-Studie von Nachemson und Petersen [23], als auch bei BRAIST [30]). Demgegenüber liegen die Erfolgsraten bei Chêneau-Versorgungen zwischen 50 und 90 % [42], was einerseits die Auswahl einer geeigneten Studie erschwert. Andererseits folgen die meisten Studien zur Chêneau-Versorgung nicht den Einschlusskriterien der SRS und sind dadurch kaum vergleichbar. Daher haben wir uns für BRAIST [30] als Kontrollgruppe entschieden.

Die Kontrollgruppe (*n* = 146) aus der BRAIST-Studie [30] hatte die folgenden Einschlusskriterien:

- adoleszente idiopathische Skoliosen
- Alter 10–15 Jahre
- Cobb-Winkel 20–40°

Das Durchschnittsalter betrug 12,7 Jahre, der durchschnittliche Krümmungswinkel betrug 30,7°, mit einer Erfolgsrate von 72 %. Der durchschnittliche Beobachtungszeitraum betrug 24,2 Monate, die erzielten Krümmungskorrekturen im Korsett sind nicht angegeben. Erfolgskriterium war eine Krümmung von <50° bei Behandlungsabschluss. Daher haben wir alternativ für die vergleichbare Gruppe III aus unserer Untersuchung auch die Ergebnisse für dieses Erfolgskriterium ermittelt.

Da es sich bei der vorliegenden Untersuchung um ein Chart Review handelt, fand zur Auswertung der Ergebnisse keine Verblindung statt. Die Messungen von Cobb-Winkel und Rumpfrotation erfolgten ausschließlich durch den Erstautor.

### Statistische Methoden

Zum statistischen Vergleich unserer Ergebnisse mit der BRAIST-Studie wurde der von Goldberg und Mitarbeitern vorgeschlagene z‑Test zum Vergleich unterschiedlicher Proportionen angewendet [7, 9]. Gruppengrößen ab *n* = 30 werden für diesen Test als optimal angesehen, allerdings werden kleinere Gruppen nicht als Ausschlusskriterium für diesen Test angesehen [7], zumal *n* als zweiter Parameter neben der Stichprobenproportion (%) das Signifikanzniveau des Ergebnisses direkt beeinflusst.

Zusätzlich wurde auch der Winkel der Rumpfrotation [5] („angle of trunk rotation“ [ATR]) zu Beginn und am Ende der Behandlung mithilfe des Skoliometers nach Bunnel für den Thorakalbereich als auch für den Lumbalbereich ermittelt. Die Veränderungen dieser Winkel wurden mithilfe des t‑Tests auf statistische Signifikanz geprüft.

Die Ergebnisse der untersuchten Patientinnengruppen wurden zuvor auf Normalverteilung untersucht (Shapiro-Wilk-Test/alpha = 0,05).

Der technische Fehler (Variabilität der Messungen) der Cobb-Winkel-Messung liegt zwischen 3 [28] und 6° [17]. Daher nimmt man in wissenschaftlichen Untersuchungen erst ab einer Winkeländerung von 6° und mehr eine tatsächliche Änderung an. Messunterschiede von ±5° werden als unverändert bewertet.

Die Messung des ATR mit dem Skoliometer hat eine hohe Reliabilität [22]. Der durchschnittliche technische Fehler liegt bei etwa 1° [22, 31].

## Ergebnisse

Die Veränderungen des durchschnittlichen Cobb-Winkels (vor Behandlung/im Korsett/bei Behandlungsabschluss) sind für alle 4 Gruppen in Tab. 2 dargestellt.

Die Korrektureffekte im Korsett liegen je nach Gruppe zwischen 47 und 52 % des Ausgangswertes und waren im t‑Test in allen Gruppen hochsignifikant.

Die Erfolgsraten lagen zwischen 86 und 88 %, bei Anwendung der Erfolgskriterien der BRAIST-Studie [17] in der vergleichbaren Gruppe III bei 96 % (Tab. 4). Die Ergebnisse sind für die Gruppen I–IV in der Tab. 5 aufgegliedert.

**Table 4:** 

	*n*	Korrektureffekt (in %)	*p*	Erfolgsrate (in %)
Gruppe I	87	47	<0,01	86
Gruppe II	39	48	<0,01	87
Gruppe III	24	52	<0,01	88/96^a^
Gruppe IV	17	48	<0,01	88
BRAIST [30]	146	Keine Angabe	–	72

Die Korrektureffekte in der CAD-Chêneau-Orthese waren im t‑Test hochsignifikant, ^a^bei Anwendung der Erfolgsparameter der BRAIST(Bracing of Adolescent Idiopathic Scoliosis Trial)-Studie (am Ende <50°)

**Table 5:** 

	*n*	Verschlechtert	Gleich	Verbessert
Gruppe I	87	12 (14 %)	42 (48 %)	33 (38 %)
Gruppe II	39	5 (13 %)	18 (46 %)	16 (41 %)
Gruppe III	24	3 (13 %)	11 (46 %)	10 (42 %)
Gruppe IV	17	2 (12 %)	7 (41 %)	8 (47 %)

Für jede Rubrik wird die Anzahl der Patientinnen und die Prozentwerte angegeben

Die Ergebnisse der statistischen Auswertung sind in Tab. 6 zu finden.

**Table 6:** 

	*n*	Erfolgsquote (in %)	z‑Wert	*p*
Gruppe I	87	86	2,58	0,01
Gruppe II	39	87	1,96	0,05
Gruppe III	24	88	1,96	0,05
Gruppe III^a^	24	96	2,58	0,01
Gruppe IV	17	88	Ns	Ns
BRAIST [30]	146	72	–	–

Gruppen I–IV wurden gegen die Ergebnisse der BRAIST(Bracing of Adolescent Idiopathic Scoliosis Trial)-Studie getestet, ^a^bei Anwendung der Erfolgsparameter der BRAIST-Studie (am Ende <50 °Cobb)

Im Vergleich zur BRAIST-Studie [30] sind die Erfolgsraten unserer Studie in den Gruppen I–III signifikant besser. Die Erfolgsquote der Gruppe IV unterscheidet sich nicht wesentlich von denen der Gruppen I–III. Die fehlende Signifikanz ist hier auf die geringe Gruppengröße zurückzuführen (*n* = 17).

Der thorakale ATR verringerte sich in der Gesamtgruppe (Gruppe I) von 10,5 auf 8,6° (*p* < 0,05), der lumbale von 6,4 auf 3,7° (*p* > 0,01).

## Diskussion

Die BRAIST-Studie [30] hat relativ lockere Erfolgskriterien. Wenn eine Krümmung die 50°-Grenze nicht erreichte oder übertraf, wurde der Verlauf als erfolgreich bezeichnet. Dies bedeutet, dass diese Studie eine nicht näher bezeichnete Anzahl von Patienten beinhaltete, welche sich zwar um 6° und mehr verschlechtert, die 50°-Grenze jedoch nicht erreicht hatten. Dementsprechend kann man davon ausgehen, dass sich bei Berücksichtigung unserer Erfolgskriterien (keine Verschlechterung von 6° und mehr) in der BRAIST-Studie [30] eine weit geringere Erfolgsrate ergeben würde. Allerdings gibt es ansonsten keine andere Vergleichsgruppe aus einer Studie hoher Evidenz, welche sich an den SRS-Kriterien [25] für Studien zur Korsettversorgung orientieren würde (Diagnose einer idiopathischen Adoleszentenskoliose, Alter 10–14 Jahre, Risser 0–2, Cobb-Winkel 25–40°). Da in der BRAIST-Studie [30] auch Jungen eingeschlossen waren, wurde entsprechend der späteren Ausreifung die Altersspanne auf 10–15 Jahre erweitert.

Interessanterweise sind nicht nur die Ergebnisse der Gruppen I–III signifikant besser als die Ergebnisse der BRAIST-Studie [30] mit den lockereren Erfolgskriterien, auch die Gruppe mit Krümmungen von 40° und mehr lag im Ergebnis mit 88 % deutlich besser als die BRAIST-Studie (20–40°) [30], obwohl Krümmungen zwischen 40 und 64° in der Gruppe enthalten waren.

Auch der Winkel der Rumpfrotation (ATR) hat sich in der Gesamtgruppe (Gruppe I) signifikant verbessert. Dies bedeutet, dass sich auch die kosmetisch bedeutsamen Parameter mit dem GBW verbessern lassen. Für die Patientinnen mag dies wichtiger sein als die Veränderungen des Cobb-Winkels, der sich am Ende statistisch nicht signifikant verbessert hat.

Die Ergebnisse unserer Untersuchung sind für die einzelnen Untergruppen, selbst unter Einschluss der Gruppe IV mit Krümmungen über 40°, recht homogen. Dies kann wohl auf die standardisierte CAD/CAM-Versorgung zurückgeführt werden. In Anbetracht dieser überdurchschnittlichen Ergebnisse sind Erfolgsraten von weniger als 70 %, wie bei einigen Studien mit dem Chêneau-Korsett [4, 27, 44] oder mit Nachtorthesen [8], aus Sicht der Betroffenen nicht akzeptabel. Allerdings sind die Erfolgsraten in unterschiedlichen Studien zur Chêneau-Versorgung sehr variabel und liegen zwischen 50 und 90 % [4, 13, 14, 16, 19, 20, 24, 32, 37, 40, 44], wobei lediglich die Ergebnisse der Studie von Korovessis et al. [14] mit denen unserer Untersuchung vergleichbar waren. Zu komplex ist die optimale Planung und Modellierung einer Chêneau-Korsettversorgung, als dass sie von jedem Techniker und für jedes mögliche Krümmungsmuster nach einem kurzen Kurs leicht beherrscht werden könnte. Zudem ist durch die relativ geringe Prävalenz der behandlungsbedürftigen Skoliosen (ca. 0,5 %) in der Gesamtbevölkerung [1, 10, 15] die Möglichkeit begrenzt, in kürzerer Zeit Erfahrungen zu sammeln und die eigenen Fähigkeiten stetig zu verbessern.

Die Betroffenen erleiden durch jedwede Korsettversorgung mehr oder weniger bedeutsame Beeinträchtigungen der Lebensqualität. Diese Beeinträchtigung muss von einer bestmöglichen Erfolgsaussicht begleitet werden.

Daher sollte die Korsettversorgung von Patienten mit Wirbelsäulendeformitäten nur standardisierten und qualitätsgesicherten CAD/CAM-Versorgungen oder sehr erfahrenen Orthopädietechnikern mit einer entsprechen hohen Fallzahl und nachgewiesener Ergebnisqualität vorbehalten bleiben. Individuelle, teils wenig erfolgversprechende Versorgungen rechtfertigen den zeitlichen Einsatz und die Beeinträchtigungen der Betroffenen nicht.

**Figure 3:**
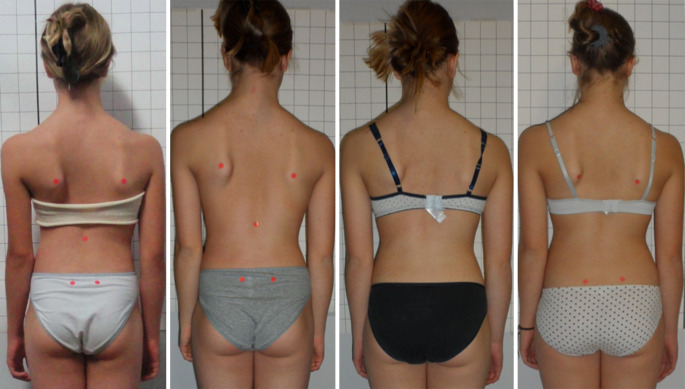

**Figure 4:**
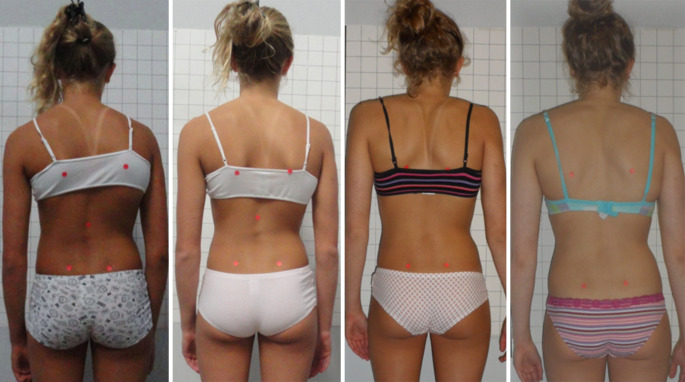

**Figure 5:**
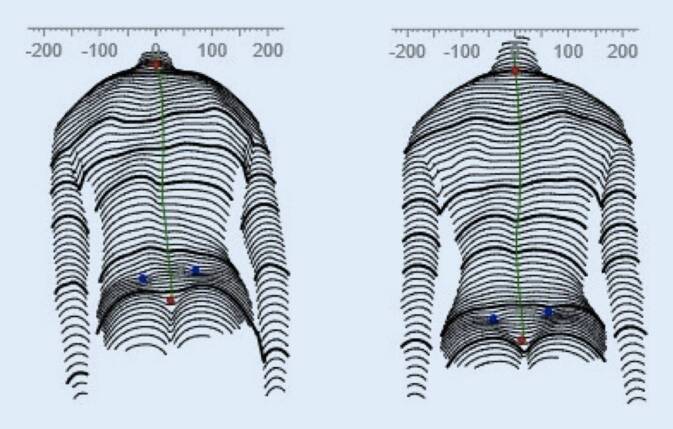

Die Studien zur Korsettversorgung bei Skoliosen fokussieren fast alle ausschließlich auf den Cobb-Winkel, obwohl dieser für die betroffenen Patienten mit idiopathischer Adoleszentenskoliose nur von untergeordneter Bedeutung ist. In dieser zahlenmäßig größten Patientengruppe mit Skoliosen sind schwerwiegende Gesundheitsbeeinträchtigungen selbst bei unbehandelten Patienten die Ausnahme [1, 29, 38]. Daher sollte auch – oder gar in erster Linie – untersucht werden, welche Korsette in der Lage sind, die Rumpfasymmetrie positiv zu beeinflussen. Ein Parameter zur Messung der Rumpfasymmetrie ist der ATR, gemessen mit dem Skoliometer nach Bunnel [5]. Allerdings beschreibt dieser Wert lediglich die Rumpfasymmetrie bei vorgebeugtem Rumpf. Auch wenn sich dieser Wert nur wenig verbessert, kann die Verbesserung der Rumpfasymmetrie im aufrechten Stand sehr deutlich ausfallen (Abb. 3). Es gibt sogar Fälle ohne wesentliche Verbesserung des Cobb-Winkels, aber mit sehr bedeutsamen Verbesserungen der Rumpfasymmetrie (Abb. 4; [36]). Grade für Patienten mit idiopathischer Adoleszentenskoliose sollte mehr Wert auf Studien gelegt werden, welche auf die kosmetisch bedeutsamen klinischen Parameter fokussieren. Hilfreich hierfür könnte auch die Oberflächenvermessung sein bei Auswertung der bildhaften Darstellung sowie der ausgegebenen Messparameter (Abb. 5).

**Figure 6:**
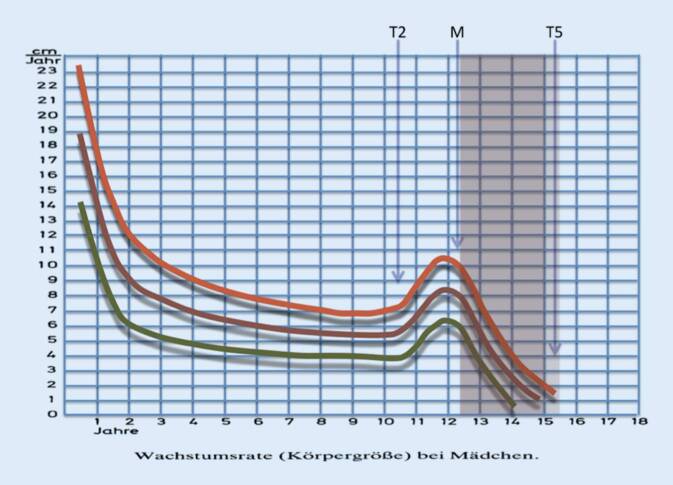

Die Patientinnen der Gruppe I hatten eine minimale Beobachtungszeit von 18 Monaten, in dieser übergeordneten Gruppe sind allerdings auch die Patientinnen der Gruppen II–IV mit Behandlungsabschluss enthalten. Natürlich kann man für die noch nicht abgeschlossenen Patientinnen keine abschließende Beurteilung treffen. Betrachtet man allerdings die Durchschnittspatientin aus dieser Gruppe, so befindet sich diese Patientin zu Behandlungsbeginn noch im Hochrisikobereich des Wachstumsschubes ([1, 10, 15, 18]; Tab. 1), während dieselbe Patientin nach mehr als 18 Monaten nur noch ein geringes Restwachstum aufweist und eine Krümmungsprogression damit weniger wahrscheinlich ist (Abb. 6). Berücksichtigt man zusätzlich, dass die Durchschnittspatientin nach Lonstein und Carlson [18] einen Progressionsfaktor von mehr als 3 und somit eine Progressionswahrscheinlichkeit von nahezu 100 % hat, so kann man schon den Ergebnissen der noch nicht abgeschlossenen Patientinnen nach einem Follow-up von mehr als 18 Monaten eine gewisse Evidenz zubilligen.

Ist man früher grundsätzlich davon ausgegangen, dass bis 2 Jahre nach Behandlungsabschluss eine zuvor erreichte Korrektur wieder zurückfällt, rechnet man heute bei Anwendung hochkorrigierender Rumpforthesen nicht mehr mit einer signifikanten Verschlechterung des Endergebnisses im Langzeitverlauf. Aulisa und Mitarbeiter haben mehr als 10 Jahre nach Korsettabschulung bleibende Korrekturen nachgewiesen, die sich nicht wesentlich von den Ergebnissen direkt nach Abschulung unterschieden [2].

Interessant ist auch, dass der Beobachtungszeitraum in der BRAIST-Studie [30] mit 24,2 Monaten deutlich kürzer war, als der unserer abgeschulten Gruppe II (36,8 Monate). Dies ist wohl einerseits darauf zurückzuführen, dass die Reduzierung der Tragezeit am Ende der Wachstumsphase in unserem Kollektiv in aller Regel über 18 Monate erfolgte, andererseits darauf, dass wir die Reduzierung der Tragezeit bei Ausgangswinkeln jenseits der 40° Grenze regelmäßig auf etwa 24 Monate ausdehnen. Das in der Kontrollgruppe [30] verwendete Boston-Brace wird in aller Regel über einen kürzeren Zeitraum abgeschult.

In einigen Studien findet man die Beobachtung, dass die reiferen Patienten, also die mit ohnehin geringerem Risiko, die geringste Progressionsneigung haben [27, 42]. Diese Beobachtung kann in Studien mit hochkorrigierenden Korsetten nicht bestätigt werden [2, 3]. Die Anzahl der progredienten Fälle in unserer Untersuchung ist zu gering, um eine Korrelation mit dem Risser-Stadium zu erstellen. Die progredienten Fälle der Gruppe II (abgeschlossenen Behandlungsfälle) finden sich in Tab. 7, aufgeschlüsselt nach dem Risser-Stadium.

**Table 7:** 

	*n*	Davon progredient
Risser 0	22	*n* = 3
Risser 1	5	*n* = 1
Risser 2	12	*n* = 1

^a^Abgeschlossene Fälle (Gruppe II)

In einer aktuellen Untersuchung [27] fand sich bei Patienten mit Krümmungen zwischen 40 und 60° eine Erfolgsquote von lediglich 40 %. Bei Berechnung des Progressionsrisikos wurde diese Quote als erfolgreich bewertet. Diese Quote ist allerdings deutlich geringer als in unserem Kollektiv mit Patientinnen der Gruppe IV (40° und mehr). Die Autoren geben an, dass die unreiferen Patienten eher progredient waren als die reiferen. Einem aktuellen Review entsprechend deutet dieser Umstand eher darauf hin, dass die Korsettqualität in der genannten Studie nicht den aktuellen Möglichkeiten entsprach [42]. Die Korrektureffekte im Korsett nehmen im Allgemeinen mit zunehmender Reife ab [3, 34]. Da eine klare Abhängigkeit des Endresultats vom erzielten Korrektureffekt im Korsett besteht [16, 26], sind die Endergebnisse bei qualitativ hochwertiger Korsettversorgung bei den unreiferen Patienten am besten, da deren Krümmungen am besten korrigiert werden können. Eine weitere Studie mit Patienten mit größeren Krümmungswinkeln scheint unsere Ergebnisse zu bestätigen. Auch in dieser Studie war mit qualitativ hochwertigen Korsetten eine hohe Erfolgsrate erzielt worden [3]. Diese Ergebnisse sprechen für die Notwendigkeit einer Vereinheitlichung des Versorgungsstandards mit korrigierenden Rumpforthesen für Patienten mit Skoliosen.

## Limitationen

Eine Schwäche unserer Untersuchung ist die geringe Zahl der abgeschlossenen Behandlungsfälle. Leider kamen viele Patientinnen erst relativ spät mit Risser-Stadien von 3 und 4 in unsere Abteilung. Viele Fälle werden ja erst nach dem Hauptwachstumsschub entdeckt, in einer Phase, da der größte Teil der Progredienz bereits stattgefunden hat. Dieser Umstand spricht für die Einführung eines regelmäßigen Schoolscreenings, beginnend mit dem Auftreten der ersten Reifezeichen.

Eine weitere Schwäche unserer Untersuchung ist das Studiendesign (Chart Review). Weitere Untersuchungen mit Studiendesigns höherer Evidenz sind sicherlich sinnvoll, um die in unserer Untersuchung gefundenen Ergebnisse zu untermauern.

## Fazit für die Praxis

- Gute Korrektureffekte im Korsett scheinen zu guten Endresultaten zu führen.
- Bei Ablehnung der Operation kann möglicherweise mit geeigneten Korsetten auch jenseits der 40°-Grenze mit dem Aufhalten der Krümmungszunahme gerechnet werden.
- Die Behandlungssicherheit für die Patienten sollte verbessert werden. Ein Ansatz hierzu kann die Verwendung standardisierter CAD(„computer aided design“)-Bibliotheken sein.
- Weitere Untersuchungen mit Studiendesigns höherer Evidenz sind notwendig, um die in unserer Untersuchung gefundenen Ergebnisse zu untermauern.

## Abkürzungen

ATR: „Angle of trunk rotation“
BRAIST: Bracing of Adolescent Idiopathic Scoliosis Trial
CAD: „Computer aided design“
CAM: „Computer aided manufacturing“
GBW: Gensingen-Brace
SD: Standardabweichung
SRS: Scoliosis Research Society
